# Expression of prostate-specific antigen (PSA) correlates with poor response to tamoxifen therapy in recurrent breast cancer

**DOI:** 10.1038/sj.bjc.6690142

**Published:** 1999-02

**Authors:** J A Foekens, E P Diamandis, H Yu, M P Look, M E Meijer-van Gelder, J G M Klijn

**Affiliations:** Division of Endocrine Oncology (Department of Medical Oncology), Mount Sinai Hospital, Toronto, Ontario, Canada; Department of Pathology and Laboratory Medicine, Mount Sinai Hospital, University of Toronto, Toronto, Ontario, Canada; Diagnostic Systems Laboratories, Webster, Texas, USA; Department of Statistics, Rotterdam Cancer Institute (Daniel den Hoed Kliniek), Academic Hospital Rotterdam, Rotterdam, The Netherlands

**Keywords:** prostate-specific antigen, breast cancer prognosis, response to therapy

## Abstract

Prostate-specific antigen (PSA) is a serine protease which may play a role in a variety of cancer types, including breast cancer. In the present study, we evaluated whether the level of PSA in breast tumour cytosol could be associated with prognosis in primary breast cancer, or with response to tamoxifen therapy in recurrent disease. PSA levels were determined by enzyme-linked immunosorbent assay (ELISA) in breast tumour cytosols, and were correlated with prognosis in 1516 patients with primary breast cancer and with response to first-line tamoxifen therapy in 434 patients with recurrent disease. Relating the levels of PSA with classical prognostic factors, low levels were more often found in larger tumours, tumours of older and post-menopausal patients, and in steroid hormone receptor-negative tumours. There was no significant association between the levels of PSA with grade of differentiation or the number of involved lymph nodes. In patients with primary breast cancer, PSA was not significantly related to the rate of relapse, and a positive association of PSA with an improved survival could be attributed to its relationship to age. In patients with recurrent breast cancer, a high level of PSA was significantly related to a poor response to tamoxifen therapy, and a short progression-free and overall survival after start of treatment for recurrent disease. In Cox multivariate analyses for response to therapy and for (progression-free) survival, corrected for age/menopausal status, disease-free interval, site of relapse and steroid hormone receptor status, PSA was an independent variable of poor prognosis. It is concluded that the level of PSA in cytosols of primary breast tumours might be a marker to select breast cancer patients who may benefit from systemic tamoxifen therapy. © 1999 Cancer Research Campaign


*British Journal of Cancer* (1999) **79**(5/6), 888–894
© 1999 Cancer Research Campaign
Article no. bjoc.1998.0142

### Expression of prostate-specific antigen (PSA) correlates with poor response to tamoxifen therapy in recurrent breast cancer

**JA Foekens[1], EP Diamandis[2], H Yu[3], MP Look[1], ME Meijer-van Gelder[1], WLJ van Putten[4] and JGM Klijn[1]**

[1]Division of Endocrine Oncology (Department of Medical Oncology), and the [2]Department of Pathology and Laboratory Medicine, Mount Sinai Hospital, Toronto, Ontario, Canada; and Department of Laboratory Medicine and Pathobiology, University of Toronto, Ontario, Canada; [3]Diagnostic Systems Laboratories, Webster, Texas, USA; [4]Department of Statistics, Rotterdam Cancer Institute (Daniel den Hoed Kliniek), Academic Hospital Rotterdam, Rotterdam, The Netherlands

**Summary** Prostate-specific antigen (PSA) is a serine protease which may play a role in a variety of cancer types, including breast cancer. In the present study, we evaluated whether the level of PSA in breast tumour cytosol could be associated with prognosis in primary breast cancer, or with response to tamoxifen therapy in recurrent disease. PSA levels were determined by enzyme-linked immunosorbent assay (ELISA) in breast tumour cytosols, and were correlated with prognosis in 1516 patients with primary breast cancer and with response to first-line tamoxifen therapy in 434 patients with recurrent disease. Relating the levels of PSA with classical prognostic factors, low levels were more often found in larger tumours, tumours of older and post-menopausal patients, and in steroid hormone receptor-negative tumours. There was no significant association between the levels of PSA with grade of differentiation or the number of involved lymph nodes. In patients with primary breast cancer, PSA was not significantly related to the rate of relapse, and a positive association of PSA with an improved survival could be attributed to its relationship to age. In patients with recurrent breast cancer, a high level of PSA was significantly related to a poor response to tamoxifen therapy, and a short progression-free and overall survival after start of treatment for recurrent disease. In Cox multivariate analyses for response to therapy and for (progression-free) survival, corrected for age/menopausal status, disease-free interval, site of relapse and steroid hormone receptor status, PSA was an independent variable of poor prognosis. It is concluded that the level of PSA in cytosols of primary breast tumours might be a marker to select breast cancer patients who may benefit from systemic tamoxifen therapy.

**Keywords:** prostate-specific antigen; breast cancer prognosis; response to therapy

Prostate-specific antigen (PSA), which is a serine protease with trypsin- and chymotrypsin-like activities (Lilja, 1985), is a useful serum marker in the management of prostate cancer (Stamey et al, 1987; Catalona et al, 1991; Oesterling, 1991). However, there is a growing body of evidence indicating that PSA may also play a role in a variety of other cancers (Levesque et al, 1995*a*; Diamandis, 1996), including breast (Giai et al, 1995; Yu et al, 1995*a*, 1996*a*, 1998; Lehrer et al, 1996; Melegos et al, 1997) and ovarian cancer (Yu et al, 1995*b*). In breast cancer tissues, the levels of PSA protein have been shown to be correlated with the levels of the progesterone receptor (PgR), rather than with those of the oestrogen receptor (ER) (Yu et al, 1994*a*). In addition, the expression of PSA in breast cancer appeared to be under control of androgens and progestins (Yu et al, 1994*b*; Zarghami et al, 1997), and it has been suggested that PSA may act as a negative growth regulator in hormone-dependent breast cancer (Lai et al, 1996).

Insulin-like growth factor I and II (IGF-I and -II) are known as potent mitogens for breast cancer cells (Osborne et al, 1989; Cullen et al, 1990). Binding of IGFs to their binding proteins (IGFBPs) can reduce their mitogenic response (Figueroa and Yee, 1992). PSA has been shown to be able to cleave IGFBP-3 (Cohen et al, 1992), suggesting that PSA may increase the bioavailability of IGFs through (local) digestion of IGFBP-3 (Kanety et al, 1993; Cohen et al, 1994) thus enhancing breast tumour growth. Human breast tumour biopsies contain a variety of IGFBPs (Manni et al, 1994; Yu et al, 1996*b*), and their levels may be of prognostic significance (Yee et al, 1994). Treatment of breast cancer patients with tamoxifen reduced serum levels of IGF-I and increased the levels of some, but not all, IGFBPs (Pollak et al, 1990; Lonning et al, 1992; Lahti et al, 1994). These results suggest that tamoxifen may regulate IGF action at the cellular level such that tumour growth promotion is inhibited. However, disruption of the interaction between IGFs and IGFBPs through proteolysis by secreted serine proteases (Campbell et al, 1992; Lalou et al, 1994), including PSA (Cohen et al, 1992), may locally disturb the balance, resulting in increased tumour cell growth through the release of active IGFs (Blat et al, 1994).

In the present study, we have investigated a possible relationship between the tumour level of PSA and response and (progression-free) survival in a large series of 434 patients with recurrent breast cancer who received tamoxifen therapy. In addition, we have studied the relationship between the tumour level of PSA and the length of relapse-free and overall survival in 1516 patients with primary breast cancer.

*Received 16 February 1998*
*Revised 15 May 1998*
*Accepted 4 June 1998*

*Correspondence to:* JA Foekens, Josephine Kefkens Institute, Dr Molewaterplein 50, 3015 GE Rotterdam, The Netherlands

**Table 1** Relationship of PSA to patient and tumour characteristics

| Characteristic | n[a] | PSA (pg mg$^{-1}$ protein) ≤ 7.25 (%) | > 7.25–20.3 (%) | > 20.3–76 (%) | > 76 (%) | *P*-value |
|---|---|---|---|---|---|---|
| All patients | 1516 | 25 | 25 | 25 | 25 | |
| Age (years) | | | | | | < 0.0001[b] |
| ≤ 40 | 189 | 18 | 27 | 24 | 31 | |
| 40–55 | 547 | 22 | 20 | 28 | 30 | |
| 56–70 | 522 | 27 | 27 | 23 | 22 | |
| > 70 | 258 | 32 | 29 | 24 | 16 | |
| Menopausal status | | | | | | |
| Premenopausal | 618 | 18 | 22 | 27 | 33 | |
| Post-menopausal | 898 | 30 | 27 | 24 | 20 | 0.0001[c] |
| Tumour size | | | | | | < 0.001[c] |
| T1 | 626 | 22 | 25 | 26 | 27 | |
| T2 | 720 | 27 | 25 | 23 | 25 | |
| T3/4 | 170 | 29 | 23 | 28 | 19 | |
| Nodal status | | | | | | ns[b] |
| N0 | 708 | 23 | 25 | 26 | 26 | |
| N1–3 | 387 | 26 | 25 | 22 | 27 | |
| N> 3 | 407 | 28 | 24 | 27 | 21 | |
| Grade | | | | | | ns[b] |
| Well | 26 | 27 | 19 | 19 | 35 | |
| Moderate | 254 | 21 | 23 | 28 | 27 | |
| Poor | 860 | 27 | 25 | 24 | 24 | |
| ER positive | | | | | | |
| No | 329 | 35 | 25 | 23 | 16 | |
| Yes | 1185 | 22 | 25 | 25 | 27 | ns[a] |
| PgR positive | | | | | | |
| No | 426 | 35 | 24 | 26 | 15 | |
| Yes | 1054 | 21 | 25 | 25 | 29 | <0.0001[a] |

[a]Because of missing values, the numbers do not always add up to 1516. [b]Spearman rank correlation. [c]Wilcoxon rank sum/Kruskal–Wallis test, including a Willcoxon-type test for trend across ordered groups.

### MATERIALS AND METHODS

### Patients and tissues

Analysis of relapse-free and overall survival was performed on 1516 patients with primary operable breast cancer diagnosed between 1978 and 1990. Inclusion criteria for patients, whose tumour or cytosol samples were stored in our tumour bank (liquid nitrogen), were as described previously (Van Putten et al, 1996). Median age of the patients at the time of surgery (modified mastectomy, 757 patients; breast conserving lumpectomy, 759 patients) was 56 years (range 24–89 years). Radiotherapy was given to 87% of the patients: on the breast/thoracic wall in 1083 patients and/or on the axilla in 511 patients, and/or on lymph node areas other than the axilla in 621 patients. Adjuvant chemotherapy (mainly CMF; cyclophosphamide, methotrexate, 5-fluorouracil) was given to 245 patients, whereas 100 patients received adjuvant hormonal therapy either alone (83 patients) or in combination with CMF (17 patients). All patients were routinely examined every 3–6 months during the first 5 years of follow-up, and once a year thereafter. Of the 1516 patients included in the study for analysis of relapse-free and overall survival, 718 (47%) showed evidence of relapse during follow-up and count as failures in the analysis for relapse-free survival. Eighty-six patients died without evidence of disease and were censored at last follow-up in the analysis for relapse-free survival. Four hundred and ninety-seven patients died after a previous relapse. A total of 583 (86 + 497) patients (38%) were considered as failures in the analysis for overall survival. The median follow-up of patients alive ($n$ = 933) is 85 months (range 13–202 months). The characteristics of the patients with respect to tumour size, lymph node status, differentiation grade of the tumour, age and menopausal status, and ER and PgR at time of surgery are listed in Table 1.

In analysis of response to first-line tamoxifen therapy for recurrent disease, 347 out of 718 patients who showed a relapse, as reported above, were evaluable according to the following criteria: the patients must have developed recurrent disease and be subjected to first-line tamoxifen therapy (40 mg day$^{-1}$). Furthermore, the patients must not have had adjuvant hormonal therapy, neoadjuvant therapy, or prior chemotherapy for advanced disease. This subset was extended with another 87 patients using the same inclusion criteria, to establish a larger series of metastatic breast cancer patients (434 patients) who received tamoxifen therapy as first-line treatment for recurrent disease.

The median age of the patients at start of treatment for advanced disease with tamoxifen was 61 years (range 28–87 years). Twenty-three per cent ($n$ = 100) of the patients were premenopausal, and

© Cancer Research Campaign 1999

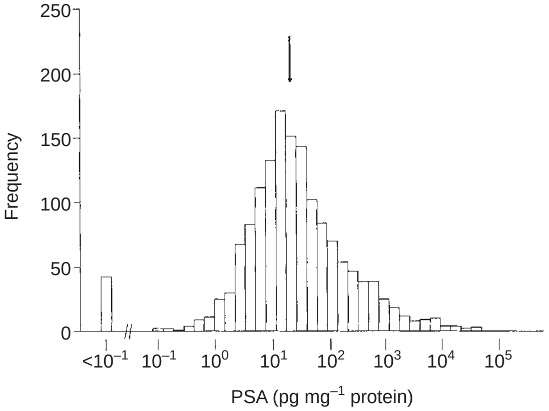

**Figure 1** Distribution of PSA over 1516 primary breast tumour cytosols. Arrow indicates the median value of 20.3 pg mg$^{-1}$ protein

**Table 2** Cox multivariate analysis for relapse-free survival for the basic model[c]

| Variable | RHR[a] | 95% CI[b] | *P*-value |
|---|---|---|---|
| Age and menopausal status | | | < 0.0001 |
| Age premenopausal | 0.66 | 0.56–0.78 | |
| Age post-menopausal | 0.90 | 0.81–1.01 | |
| Post- vs premenopausal | 1.82 | 1.37–2.41 | |
| Tumour size | | | <0.0001 |
| T2 vs T1 | 1.44 | 1.21–1.72 | |
| T3/4 vs T1 | 1.89 | 1.47–2.41 | |
| Nodal status | | | |
| N1–3 vs N0 | 1.51 | 1.24–1.84 | <0.0001 |
| N>3 vs N0 | 2.96 | 2.45–3.57 | |
| + ER[d] | | | |
| Positive vs negative[e] | 0.92 | 0.77–1.10 | 0.36 |
| Continuous | 0.99 | 0.95–1.02 | 0.45 |
| + PgR[d] | | | |
| Positive vs negative[e] | 0.86 | 0.73–1.02 | 0.08 |
| Continuous | 0.97 | 0.93–1.00 | 0.05 |
| + PSA[d] | | | 0.97 |
| Q2 vs Q1[f] | 1.01 | 0.82–1.24 | |
| Q3 vs Q1 | 1.01 | 0.82–1.25 | |
| Q4 vs Q1 | 0.97 | 0.78–1.20 | |
| Continuous | 1.01 | 0.97–1.05 | 0.69 |

[a]RHR, relative hazard rate. [b]95% CI, 95% confidence interval. [c]The basic model included age/menopausal status, tumour size and nodal status. [d]Added separately to the basic model. [e]Cut-off points used: 10 fmol mg$^{-1}$ protein for both ER and PgR. [f]'Q1': 0–7.25 pg mg$^{-1}$ protein; 'Q2': >7.25–20.3 pg mg$^{-1}$ protein, 'Q3': >20.3–76 pg mg$^{-1}$ protein; 'Q4': > 76 pg mg$^{-1}$ protein.

77% ($n$ = 334) post-menopausal. The dominant site of disease was visceral in 157 patients, bone in 222 patients, and soft tissue in 55 patients. Twenty-nine patients (7%) had metastatic disease (M1 patients) at time of primary surgery. After primary surgery, 83 patients (19%) had received systemic adjuvant chemotherapy (CMF, 65 patients; FAC; 5-fluorouracil, adriamycin, cyclophosphamide, 18 patients). During their life with metastatic disease, 60.4% of the patients were subsequently treated with one or more additional hormonal treatments (mostly progestins) after progression on the first-line tamoxifen treatment. After occurrence of resistance to hormonal therapy, 226 patients (52.1%) received additional chemotherapy (mainly CMF). Ninety-two patients are still alive at time of analysis with a median follow-up of 81 months (range 19–149 months), and 342 patients have died with a median survival time of 41 months (range 3–166 months). During follow-up after start of first-line endocrine therapy, 91% of the patients (395 out of 434) developed tumour progression with a median time to progression of 163 days (range 18 days to >9 years). During endocrine therapy, patients were assessed at the outpatient clinic on average once every 6 weeks; during long-term remission, up to once every 12 weeks. Response to therapy was assessed by standard criteria for objective response (complete and partial remission). Patients with stable disease (SD; no change for more than 6 months) have a survival probability similar to those with a partial remission (Foekens et al, 1994). Response to treatment was defined as a patient having either an objective response or a prolonged SD with a time to treatment failure of more than 6 months, as defined previously (Nicholson et al, 1989; Ravdin et al, 1992; Foekens et al, 1994, 1995). In case of doubt, the worst category of response was chosen. Of the 434 patients who received tamoxifen therapy as first-line treatment for recurrent disease, 221 (51%) responded (55 objective response, 166 stable disease). Of the 213 patients who did not respond (49%), 45 patients had no change of disease for a period equal to or shorter than 6 months, and 168 patients showed progressive disease.

### Assay of PSA, ER and PgR

Tumour tissues were stored in liquid nitrogen and pulverized in the frozen state with a microdismembrator as recommended by the European Organization for Research and Treatment of Cancer (EORTC) for processing of breast tumour tissue for cytosolic ER and PgR determinations (EORTC Breast Cancer Cooperative Group, 1980). The resulting tissue powder was suspended in EORTC receptor buffer (10 mM dipotassium hydrogen phosphate buffer, containing 1.5 mM dipotassium chloride EDTA, 3 mM sodium azide, 10 mM monothioglycerol, and 10% v/v glycerol, pH 7.4). The suspension was centrifuged for 30 min at 100 000 ***g*** to obtain the supernatant fraction (cytosol). ER and PgR levels were determined by ligand binding assay or with enzyme immunoassay as described previously (Foekens et al, 1989). The cut-off point used to classify tumours as ER or PgR positive and negative was 10 fmol mg$^{-1}$ cytosolic protein.

Cytosolic PSA levels were determined in duplicate with a highly sensitive and specific immunofluorometric procedure, which has previously been described in detail (Ferguson et al, 1996). PSA concentrations in the tumour samples are expressed as pg mg$^{-1}$ cytosolic protein. PSA is a very stable molecule and its concentration in breast tumour cytosols does not change when the samples are kept frozen at –20°C or lower. Consequently, there were no statistically significant differences in the levels of PSA in relation to the duration of storage of the samples.

### Statistics

The strength of the associations of PSA with other variables was tested with non-parametric tests: for categorical variables, the Wilcoxon rank–sum or Kruskal–Wallis test, including a Wilcoxon-type test for trend across ordered groups when appropriate and the Spearman rank correlation ($r_s$) for continuous variables. In patients with recurrent breast cancer who received tamoxifen as first-line

© Cancer Research Campaign 1999

**Table 3** Relation of PSA status to response

| Response[a] | All patients | PSA level | | |
|---|---|---|---|---|
| | | Low[b] | Intermediate[b] | High[b] |
| No | 213 | 13 | 154 | 46 |
| Yes | 221 | 32 | 159 | 30 |
| Total | 434 | 45 | 313 | 76 |
| Fraction responders (%) | 51 | 71 | 51 | 39 |
| | | | $P$ < 0.01 | |

[a]Response, 'yes': objective response and stable disease (no change for more than 6 months); 'no': progressive disease and no change for a period equal or less than 6 months. [b]'Low', 0–2.5 pg mg$^{-1}$ protein; 'intermediate', > 2.5–125 pg mg$^{-1}$ protein; 'high', > 125 pg mg$^{-1}$ protein.

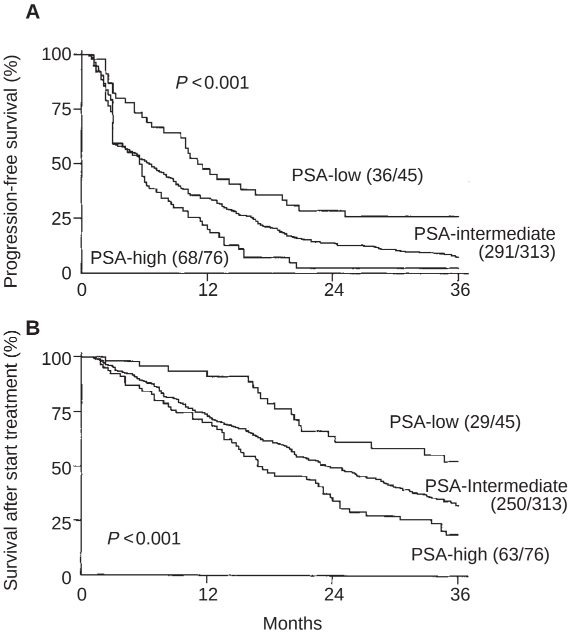

**Figure 2** Progression-free (**A**) and overall survival after start of tamoxifen treatment (**B**) in 434 patients with recurrent breast cancer who received tamoxifen as first-line treatment as a function of the level of PSA. 'High', > 125 pg mg$^{-1}$ protein; 'intermediate', > 2.5–125 pg mg$^{-1}$ protein; 'low', 0–2.5 pg mg$^{-1}$ protein. Numbers in parentheses indicate the number of failures/total number of patients in each group treatment, in a test for trend in logistic regression analysis, and Cox regression analysis, higher levels of PSA were associated with a poor response, short progression-free and overall survival after start of tamoxifen treatment respectively. This justified a search for a cut-off point(s) to allow analysis of PSA as a categorical variable. For this search, isotonic regression analysis, with all three parameters as end point, was applied (Barlow et al, 1972). Relapse-free and overall survival probabilities in primary breast cancer and progression-free survival probabilities after start of first-line therapy with tamoxifen in recurrent disease were calculated by the actuarial method of Kaplan and Meier (1958). Both uni- and multivariate analysis was performed using the Cox proportional hazard model. The associated likelihood ratio test was used to test for differences. Logistic regression analysis was applied for uni- and multivariate analysis of response.

### RESULTS

### PSA levels and relationships with patient and tumour characteristics

The levels of PSA in primary tumours of patients which were evaluable for analysis of relapse-free and overall survival ranged from 0 to 50 000 pg mg$^{-1}$ protein (median 20.3; mean ± s.d. 398 ± 2495 pg mg$^{-1}$ protein). The distribution of the levels of PSA in 1516 tumour cytosols is shown in Figure 1.

In Table 1, we present the percentage of tumours with PSA levels divided in quartiles, in relation to patient and tumour characteristics. Low levels of PSA were more often found in larger tumours, and in tumours of older and post-menopausal patients. There was no significant association between the levels of PSA and ER ($r_s$ = 0.01; $P$ > 0.05), grade of differentiation, or number of involved lymph nodes. The levels of PSA were positively correlated with those of PgR ($r_s$ = 0.16; $P$ < 0.0001). PSA levels in PgR-positive tumours were higher than in PgR-negative tumours, with respective median values of 24.7 and 13.3 pg mg$^{-1}$ protein ($P$ < 0.0001). Although the levels of PSA and ER were not significantly correlated when analysed by Spearman rank correlation, PSA levels were higher in ER-positive tumours than in ER-negative tumours (median 22.5 and 12.4 pg mg$^{-1}$ protein respectively; $P$ = 0.0001). In the four subgroups of tumours stratified by ER and PgR status, PSA levels were lowest in the ER–/PgR– and ER+/PgR– subgroups (11.7 and 16.0 pg mg$^{-1}$ protein respectively), and highest in the ER–/PgR+ and ER+/PgR+ subgroups (22.4 and 24.9 pg mg$^{-1}$ protein respectively). Thus, the data suggest, in analogy with previous observations (Yu et al, 1994*a*, 1995*a*), that PSA is associated with PgR rather than with ER.

### PSA and relapse-free and overall survival

In Cox univariate regression analysis including 1516 patients with primary breast cancer, there was a statistically significant association between log-transformed PSA values and the length of overall survival ($P$ = 0.009), but not with the length of relapse-free survival ($P$ = 0.42). The lack of a significant association between PSA and relapse-free survival did not justify a search for cut-off points for PSA. Therefore, the patients were divided into four groups with an approximately equal number of patients based on their PSA levels. In analysis for overall survival, patients with tumours containing the 25% highest levels of PSA showed a favourable prognosis with a relative hazard rate (RHR) and 95% confidence interval (95% CI) of 0.69 (0.55–0.87) compared with those with the 25% lowest PSA levels ($P$ = 0.002). In additional analyses, after correction for age only, PSA was not significantly associated with overall survival (not shown). Therefore, the observed association of PSA with improved overall survival in univariate analysis can be explained by its negative relationship with age. In univariate analysis for relapse-free survival, there was no significant difference ($P$ = 0.26) between the groups with the highest and lowest 25% PSA. Next, the association of the levels of PSA with relapse-free survival, after correction for classical prognostic factors, was studied. A basic model was introduced which included age/menopausal status, nodal status and tumour size (Table 2). When added separately to the basic model as dichotomized or continuous variables, ER and PgR were not or only marginally associated with an improved relapse-free survival. In addition, PSA, when analysed both

© Cancer Research Campaign 1999

categorically and continuously, was not independently associated with relapse-free survival (Table 2). Similar results were obtained by multivariate analysis for overall survival (not shown), except that ER and PgR significantly ($P$ < 0.0001) added to the basic model. When adjuvant hormonal and chemotherapy were added as indicator variables to the multivariate model for relapse-free survival, they were independently associated with decreased rates of relapse (RHR = 0.69, $P$ = 0.01; RHR = 0.61, $P$ < 0.0001; respectively). They did not affect the estimated regression coefficients of PSA, and were therefore not included in the model shown in Table 2. Furthermore, also in subgroups of node-negative and node-positive patients with or without adjuvant therapy, no associations were found between PSA and relapse-free survival.

### PSA and response to tamoxifen therapy

A total of 434 patients were included in the analysis of response to first-line therapy with tamoxifen for recurrent disease as a function of the level of PSA in the primary tumour. The median PSA level in these tumours (23.0 pg $mg^{-1}$ protein) was comparable to those in the tumours of the 1516 patients who were analysed for relapse-free and overall survival (20.3 pg $mg^{-1}$ protein).

Of the 434 patients who were treated with tamoxifen for recurrent disease, 221 (51%) responded. The median duration of response was 15 months. In a test for trend using logarithmically transformed PSA values, increasing levels of PSA were significantly associated with a worse response, a poor progression-free survival, and a short post-relapse survival after start of tamoxifen treatment (all, $P$ < 0.01). Isotonic regression analyses using these three end points suggested the presence of two cut-off points. Therefore, in subsequent analyses using PSA as a categorical variable, the tumours were classified as PSA-low (0–2.5 pg $mg^{-1}$ protein; 45 patients), PSA-intermediate (>2.5–125 pg $mg^{-1}$ protein; 313 patients) and PSA-high (>125 pg $mg^{-1}$ protein; 76 patients). With increasing levels of PSA, the response to tamoxifen significantly decreased from 71% in the PSA-low group, to 51% in the PSA-intermediate group, and to 39% in the PSA-high group ($P$ < 0.01) (Table 3). The fraction of complete and partial remissions decreased from 22% in the PSA-low group, via 13% in the PSA-intermediate group, to 5% in the PSA-high group. This decrease in objective response rates with increasing levels of PSA was present both in patients with ER levels ≤75 fmol $mg^{-1}$ protein (41% of the tumours) and with ER levels >75 fmol $mg^{-1}$ protein (59% of the tumours). In the ER-low group, the objective response rates decreased from 11%, via 9%, to 5%, whereas in the ER-high group the objective response rates decreased from 31% in the PSA-low group, via 16% for the PSA-intermediate group, to 5% for the PSA-high group.

Multivariate analysis for response was performed, including as variables age and menopausal status, dominant site of relapse, disease-free interval, ER and PgR (as continuous variables), and PSA (intermediate compared with low; high compared with low). Significant indicators of poor response in the final model were: a short disease-free interval (≤1 year vs >1 year; $P$ < 0.0001), visceral metastasis ($P$ = 0.04), low levels of ER ($P$ < 0.0001), and high levels of PSA. Compared with tumours with low PSA levels, those with intermediate levels [odds ratio (OR) 0.41; 95% CI 0.20–0.83; $P$ = 0.01] or high levels (OR 0.27; 95% CI 0.12–0.61; $P$ = 0.002) were significantly associated with a poor response to tamoxifen therapy. Inclusion of adjuvant chemotherapy as indicator variable in the multivariate regression models did not have an effect on the estimated regression coefficients of PSA.

### PSA and progression-free and overall survival after start of tamoxifen treatment

In analogy with the relationship of PSA and response, the length of progression-free survival on tamoxifen therapy decreased with increasing PSA levels in the three subgroups of patients stratified by PSA levels, respectively ranging from 11.0 months in PSA-low, via 6.3 months in PSA-intermediate, to 5.5 months in tumours with the highest PSA levels. The Kaplan–Meier curves for progression-free survival and overall survival after start of tamoxifen therapy as a function of PSA status are shown in Figure 2A and B respectively. In multivariate analyses for progression-free survival and for overall survival after start of tamoxifen treatment, PSA significantly contributed to the final models. In analysis for progression-free survival, younger age, a short disease-free interval, visceral metastasis, decreasing ER and PgR levels, and intermediate (RHR 1.91; 95% CI 1.33–2.74; $P$ < 0.001) and high PSA levels (RHR 2.12; 95% CI 1.38–3.26; $P$ < 0.001) were significant variables to predict an early progression. In overall survival after start of tamoxifen treatment, PSA significantly ($P$ < 0.001) contributed to the final multivariate model, including disease-free interval, visceral metastasis and ER. The respective relative hazard rates for death for tumours with intermediate and high PSA levels, compared with PSA-low tumours, were 1.98 (95% CI 1.33–2.93) and 2.48 (95% CI 1.57–3.92).

### DISCUSSION

For patients with breast cancer, it would be beneficial to have tools available, in addition to the classical prognostic factors, which could independently predict the rate of recurrence in primary breast cancer and the efficacy of response to systemic therapy. In patients with recurrent breast cancer, the presence of steroid receptors in the primary tumour does not fully predict those patients who are likely to benefit from endocrine therapy. About half of the patients with steroid receptor-positive tumours do not respond favourably to anti-oestrogen treatment. Therefore, biological factors, other than ER and PgR, might be valuable for refinement of therapy tailored to the patient's need. The aberrant expression of a specific cell biological parameter which is associated with a high risk of relapse in primary breast cancer does not necessarily justify the administration of adjuvant treatment. A particular form of systems therapy may actually be inactive or even stimulate tumour growth by triggering the cell biological factor. Therefore, biological parameters should be evaluated both for their prognostic value in primary and in metastatic breast cancer. One factor which may be helpful in assessing prognosis in primary breast cancer is the serine protease PSA (Yu et al, 1995*a*), whose expression is under the control of steroid hormones, i.e. androgens and progestins (Yu et al, 1994*b*; Zarghami et al, 1997). PSA expression may be related to response to endocrine therapy.

In the present study of 1516 breast cancer cytosols, the median PSA level was 20.3 pg $mg^{-1}$ protein. This level is identical to that reported in a series of 199 patients (Yu et al, 1996*a*), but slightly higher than the observed 15 pg $mg^{-1}$ protein determined in 1275 breast tumours which, however, were pooled from separate studies (Yu et al, 1994*a*). The slightly higher PSA levels in the present study cannot have resulted from the use of different assays to measure PSA because both series of tumours were analysed by the same method. One reason for the observed variation in PSA levels in the different series of samples could be the different composition

 © Cancer Research Campaign 1999

of the buffers which were used to prepare the tumour cytosols (Yu et al, 1994*a*, 1995*a*, 1996*a*). Despite the distinct patient groups and some technical dissimilarities, the data shown in the present study confirm those which have been published previously regarding the relationship of PSA with patient and tumour characteristics. These relationships are the observed higher PSA levels in younger patients (Yu et al, 1994*a*, 1996*a*), in smaller tumours (Yu et al, 1995*a*), and in steroid hormone receptor-positive tumours, especially in those which were positive for PgR (Yu et al, 1994*a*, 1995*a*; Levesque et al, 1995*b*). In analogy with a previous study (Yu et al, 1995*a*), we found no association between PSA and nodal status.

In the two studies on the prognostic relevance of PSA published so far, involving 174 and q53 primary breast cancer patients, a cut-off point of 30 pg $mg^{-1}$ protein was chosen to classify breast tumours as PSA positive and negative (Yu et al, 1995*a*, 1998). Patients with PSA-positive tumours showed an improved relapse-free survival in both Cox univariate and multivariate regression analyses (Yu et al, 1995*a*, 1998). In our large study, however, there was no statistically significant association between the levels of PSA in the primary tumour and the rate of relapse. We, therefore, considered it unjustified to search for a cut-off point. To enable analysis of PSA as a categorical variable, we have divided the patients in four subgroups with an equal number of patients (quartiles), based on the levels of PSA. For comparison of our data with those published previously (Yu et al, 1995*a*, 1998), with 27% and 24% respectively, of the patients classified as positive, our patients in the fourth quarter group could be considered as PSA positive. Indeed, these patients showed an improved survival in univariate analysis, but after correction for age the relationship between PSA and age was no longer statistically significant. In contrast with the previous studies (Yu et al, 1995*a*, 1998), high levels of PSA were not significantly associated with a decreased relapse rate. Compared with the study of Yu et al (1995*a*, 1998), we had a different group of patients, a longer follow-up period (median 85 vs 33 and 73 months) and, as a consequence, a higher proportion of patients who relapsed (47% vs 24% and 21%).

The present study is the first to show that high levels of PSA in primary breast tumours are associated with a poor response and a short duration of response to first-line tamoxifen therapy in recurrent disease. The association of high PSA expression with poor patient outcome was independent of age/menopausal status, disease-free interval between primary surgery and the occurrence of relapse, site of metastasis, adjuvant chemotherapy, and ER and PgR status. It is puzzling why a factor which is positively related to steriod hormone receptors is associated with a poor response and survival on tamoxifen therapy in recurrent disease. The association of PSA with poor response and survival in recurrent breast cancer may be due to its intrinsic biological properties of being a serine protease. In other words, PSA, like the serine protease urokinase-type plasminogen activator (uPA) (Foekens et al, 1995), may increase tumour growth by enzymatic activation of specific growth factor (receptor)-binding protein pathways. In its active form, uPA is able to convert plasminogen into plasmin. Plasmin can subsequently directly degrade components of the extracellular matrix, and furthermore can activate type-IV collagenase. These activated enzyme systems, in which uPA is considered to play a key role, lead to the processes of tumour cell invasion and metastasis (Danø et al, 1985; Mignatti and Rifkin, 1993; Andreasen et al, 1997). Almost a decade ago, a high level of uPA activity in primary breast tumours was found to be associated with an early relapse (Duffy et al, 1988). There is now a profound body of evidence that a higher level of uPA protein, which in contrast to PSA, is found more frequently in ER- and PgR-negative tumours (Jänicke et al, 1990), is associated with poor prognosis in primary breast cancer (Jänicke et al, 1990; Foekens et al, 1992; reviewed by: Duffy, 1996; Andreasen et al, 1997; Schmitt et al, 1997), and also in a broad variety of other cancer types (Duffy, 1996). In analogy to uPA, the relationship of PSA to a poor response to tamoxifen therapy, which does not affect PSA expression in vitro (Zarghami et al, 1997), may, for example, be due to a local increase in the concentration of bioavailable IGFs by cleaving IGFBPs (Cohen et al, 1992, 1994; Figueroa and Yee, 1992; Kanety et al, 1993) through tumour cell-secreted PSA. The free IGFs, abundantly present around the tumour cells because of the focal action of secreted PSA, are potent mitogens for breast cancer cells (Osborne et al, 1989; Cullen et al, 1990). They may disturb the balance and abolish the growth inhibitory effects induced by tamoxifen, despite a reduction in total serum levels of IGFs (Pollak et al, 1990; Lonning et al, 1992; Yee et al, 1994).

Our study suggests that a low tumour level of PSA is associated with an improved rate and duration of response, and a prolonged progression-free and overall survival after start of tamoxifen therapy for metastatic disease. Thus, low tumour levels of PSA may identify patients who will respond better to first-line tamoxifen therapy. In contrast, patients with a high tumour level of PSA might benefit from chemotherapy. However, suggestions for the most promising systemic treatment for the individual breast cancer patient based on the tumour level of PSA awaits confirmatory studies including those patients who received chemotherapy as well, preferably within prospective trials.

### ACKNOWLEDGEMENTS

Work performed at the Rotterdam Cancer Institute was supported by grant DDHK 96-1234 of the Dutch Cancer Society, and work performed at Mount Sinai Hospital by the Canadian Breast Cancer Research Initiative of the National Cancer Institute of Canada. We gratefully express our thanks to the surgeons, pathologists and internists of the St. Clara Hospital, Ikazia Hospital, St. Franciscus Gasthuis and the Rotterdan Cancer Institute (Daniel den Hoed Kliniek), all located in Rotterdam, for the supply of tumour tissues and/or for assisting us in the collection of the clinical follow-up data.

### REFERENCES

Andreasen PA, Kjøller L, Christensen L and Duffy MJ (1997) The urokinase-type plasminogen activator system in cancer metastasis: a review. *Int J Cancer* **72**: 1–22

Barlow RE, Bartelomew DJ, Bremmer JM and Brunck HD (1972) *Statistical Interference Under Order Restrictions*. John Wiley & Sons: London

Blat C, Villaudy J and Binoux M (1994) In vivo proteolysis of serum insulin-like growth factor (IGF) binding protein-3 results in increased availability of IGF to target cells. *J Clin Invest* **93**: 2226–2230

Campbell PG, Novak JF, Yanosick TB and McMaster JH (1992) Involvement of the plasmin system in dissociation of the insulin-like growth factor-binding protein complex. *Endocrinology* **130**: 1401–1412

Catalona WJ, Smith DS, Ratliff TL, Dodds KM, Coplen DE, Yuan JJ, Petros JA and Andriole GL (1991) Measurement of prostate specific antigen in serum as a screening test for prostate cancer. *N Engl J Med* **324**: 1156–1161

Cohen P, Graves HCB, Peehl DM, Kamarci M, Giundice LC and Rosenfeld RG (1992) Prostate-specific antigen (PSA) is an insulin-like growth factor binding protein-3 protease found in seminal plasma. *J Clin Endocrinol Metab* **75**: 1046–1053

Cohen P, Peehl DM, Graves HCB and Rosenfeld RG (1994) Biological effects of prostate specific antigen as an insulin-like growth factor binding protein-3 protease. *J Endocrinol* **142**: 407–415

© Cancer Research Campaign 1999

Cullen KJ, Yee D, Sly WS, Perdue J, Hampton B, Lippman ME and Rosen N (1990) Insulin-like growth factor receptor expression and function in human breast cancer cells. *Cancer Res* **50**: 48–53

Danø K, Andreasen PA, Grøndahl-Hansen J, Kristensen PI, Nielsen LS and Skriver L (1985) Plasminogen activators, tissue degradation, and cancer. *Adv Cancer Res* **44**: 139–266

Diamandis ER (1996) Prostate specific antigen – new applications in breast and other cancers. *Anticancer Res* **16**: 3983–3986

Duffy MJ (1996) Proteases as prognostic markers in cancer. *Clin Cancer Res* **2**: 613–618

Duffy MJ, O'Grady P, Devaney D, O'Siorian L, Fennelly JJ and Lijnen HJ (1988) Urokinase plasminogen activator, a marker for aggressive breast carcinomas: preliminary report. *Cancer* **62**: 531–533

EORTC Breast Cancer Cooperative Group (1980) Revision of the standards for the assessment of hormone receptors in human breast cancer. *Eur J Cancer* **16**: 1513–1515

Ferguson RA, Yu H, Kalyvas M, Zammit S and Diamandis EP (1996) Ultrasensitive detection of prostate specific antigen by a new time resolved immunofluorometric assay and the Immulite immunochemiluminescent third generation assay: potential applications in prostate and breast cancers. *Clin Chem* **42**: 675–684

Figueroa JA and Yee D (1992) The insulin-like growth factor binding proteins (IGFBPs) in human breast cancer. *Breast Cancer Res Treat* **22**: 81–90

Foekens JA, Portengen H, van Putten WLJ, Peters HA, Krijnen HLJM, Alexieva-Figusch J and Klijn JGM (1989) Prognostic value of estrogen and progesterone receptors measured by enzyme immunoassays in human breast tumor cytosols. *Cancer Res* **49**: 5823–5828

Foekens JA, Schmitt M, van Putten WLJ, Peters HA, Bontenbal M, Jänicke F and Klijn JGM (1992) Prognostic value of urokinase-type plasminogen activator in 671 primary breast cancer patients. *Cancer Res* **52**: 6101–6105

Foekens JA, Portengen H, Look MP, van Putten WLJ, Thirion B, Bontenbal M and Klijn JGM (1994) Relationship of PS2 with response to tamoxifen therapy in patients with recurrent breast cancer. *Br J Cancer* **70**: 1217–1223

Foekens JA, Look MP, Peters HA, van Putten WLJ, Portengen H and Klijn JGM (1995) Urokinase-type plasminogen activator and its inhibitor PAI-1: predictors of poor response to tamoxifen therapy in recurrent breast cancer. *J Natl Cancer Inst* **87**: 751–756

Giai M, Yu II, Roagna R, Ponzone R, Katsaros D, Levesque MA and Diamandis EP (1995) Prostate-specific antigen in serum of women with breast cancer. *Br J Cancer* **72**: 728–731

Jänicke F, Schmitt M, Hafter R, Hollreider A, Babic R, Ulm K, Gössner W and Graeff H (1990) Urokinase-type plasminogen activator (u-PA) antigen is a predictor of early relapse in breast cancer. *Fibrinolysis* **4**: 69–78

Kanety H, Madjar Y, Dagan Y, Levi J, Papa MZ, Pariente C, Goldwasser B and Karasik A (1993) Serum insulin-like growth factor-binding protein-2 (IGFBP-2) is increased and IGFBP-3 is decreased in patients with prostate cancer: correlation with serum prostate-specific antigen. *J Clin Endocrinol Metab* **77**: 229–233

Kaplan EL and Meier P (1958) Non-parametric estimation from incomplete observations. *J Am Stat Assoc* **53**: 457–481

Lahti EI, Knip M and Laatikainen TJ (1994) Plasma insulin-like growth factor I and its binding proteins 1 and 3 in postmenopausal patients with breast cancer receiving long term tamoxifen. *Cancer* **74**: 618–624

Lai LC, Erbas H, Lennard TW and Peaston RT (1996) Prostate-specific antigen in breast cyst fluid: possible role of prostate-specific antigen in hormone-dependent breast cancer. *Int J Cancer* **66**: 743–746

Lalou C, Silve C, Rosato R, Segivia B and Binoux M (1994) Interactions between insulin-like growth factor-I (IGF-I) and the system of plasminogen activators and their inhibitors in the control of IGF-binding protein-3 production and proteolysis in human osteosarcoma cells. *Endocrinology* **135**: 2318–2326

Lehrer S, Terk M, Picolli SP, Song HK, Lavagnini P and Luderer AA (1996) Reverse transcriptase-polymerase chain reaction for prostate-specific antigen may be a prognostic indicator in breast cancer. *Br J Cancer* **74**: 871–873

Levesque M, Hu H, D'Costa M and Diamandis EP (1995*a*) Prostate-specific antigen expression by various tumors. *J Clin Lab Anal* **9**: 123–128

Levesque MA, Clark GM, Yu H and Diamandis EP (1995*b*) Immunofluorometric analysis of p53 protein and prostate-specific antigen in breast tumours and their association with other prognostic indicators. *Br J Cancer* **72**: 720–727

Lilja II (1985) A kallikrein-like serine protease in prostatic fluid cleaves the predominant seminal vesicle protein. *J Clin Invest* **76**: 1899–1903

Lonning PE, Hall K, Aakvaag A and Lien EA (1992) Influence of tamoxifen on plasma levels of insulin-like growth factor I and insulin-like growth factor binding protein I in breast cancer patients. *Cancer Res* **52**: 4719–4723

Manni A, Badger B, Wei L, Zaenglein A, Grove R, Khin S, Heitjan D, Shimasaki S and Ling N (1994) Hormonal regulation of insulin-like growth factor II and insulin-like growth factor binding protein expression by breast cancer cells in vivo: evidence for stromal epithelial interactions. *Cancer Res* **54**: 2934–2942

Melegos DN, Yu H, Ashok M, Wang C, Stanczyk F and Diamandis EP (1997) Prostate-specific antigen in female serum, a potential new marker of androgen excess. *J Clin Endocrinol Metab* **82**: 777–780

Mignatti P and Rifkin DB (1993) Biology and biochemistry of proteinases in tumor invasion. *Physiol Rev* **73**: 161–195

Nicholson S, Sainsbury JRC, Halcrow P, Farndon JR, Chambers P and Harris AL (1989) Expression of epidermal growth factor receptors associated with lack of response to endocrine therapy in recurrent breast cancer. *Lancet* **i**: 182–185

Oesterling JE (1991) Prostate specific antigen: a critical assessment of the most useful tumor marker for adenocarcinoma of the prostate. *J Urol* **145**: 907–923

Osborne CK, Coronado EB, Kitten LJ, Arteaga CL, Fuqua SAW, Ramasharma K, Marschal M and Li CH (1989) Insulin-like growth factor-II: a potential autocrine/paracrine growth factor for human breast cancer acting via the IGF-I receptor. *Mol Endocrinol* **3**: 1701–1709

Pollak M, Costantino J, Polychronakos C, Blauer SA, Guyda H, Redmond C, Fisher B and Margolese R (1990) Effect of tamoxifen on serum insulin-like growth factor 1 levels in stage I breast cancer patients. *J Natl Cancer Inst* **82**: 1693–1697

Ravdin PM, Green S, Dorr TM, McGuire WL, Fabian C, Pugh RP, Carter RD, Rivkin SE, Borst JR, Belt RJ, Metch B and Osborne CK (1992) Prognostic significance of progesterone receptor levels in estrogen receptor-positive patients with metastatic breast cancer treated with tamoxifen: results of a prospective Southwest Oncology Group study. *J Clin Oncol* **10**: 1284–1291

Schmitt M, Harbeck N, Thomssen C, Wilhelm O, Magdolen V, Reuning U, Ulm K, Höfler H, Jänicke F and Graeff H (1997) Clinical impact of the plasminogen activation system in tumor invasion and metastasis: prognostic relevance and target for therapy. *Thromb Haemostasis* **78**: 285–296

Stamey TA, Yang N, Hay AR, McNeal JE, Freiha FS and Redwine E (1987) Prostate-specific antigen as a serum marker for adenocarcinoma of the prostate. *N Engl J Med* **317**: 909–916

Van Putten WLJ, Klijn JGM, Meijer-van Gelder ME, Look MP and Foekens JA (1996) Multiparameter analysis of prognostic factors in breast cancer. In *Breast Cancer Advances in Biology and Therapeutics*. Calvo F, Crepin M and Magdelenat H (eds.), pp. 209–215. John Libbey Eurotext: Montrouge

Yee D, Sharma J and Hilsenbeck SG (1994) Prognostic significance of insulin-like growth factor-binding protein expression in axillary lymph node-negative breast cancer. *J Natl Cancer Inst* **86**: 1785–1789

Yu H, Diamandis EP and Sutherland DJA (1994*a*) Immunoreactive prostate-specific antigen levels in female and male breast tumors and its association with steroid hormone receptors and patient age. *Clin Biochem* **27**: 75–79

Yu H, Diamandis ER, Zarghami N and Gras L (1994*b*) Induction of prostate-specific antigen production by steroids and tamoxifen in breast cancer cells lines. *Breast Cancer Res Treat* **32**: 291–300

Yu H, Giai M, Diamandis EP, Katsaros D, Sutherland DJA, Levesque MA, Roagna R, Ponzone R and Sismondi P (1995*a*) Prostate-specific antigen is a new favorable prognostic indicator for women with breast cancer. *Cancer Res* **55**: 2104–2110

Yu H, Diamandis EP, Levesque M, Asa SL, Monne M and Croce CM (1995*b*) Expression of the prostate-specific antigen gene by a primary ovarian carcinoma. *Cancer Res* **55**: 1603–1606

Yu H, Diamandis EP, Levesque M, Giai M, Roagna R, Ponzone R, Sismondi P, Monne M and Croce CM (1996*a*) Prostate specific antigen in breast cancer, benign breast disease and normal breast tissue. *Breast Cancer Res Treat* **40**: 171–178

Yu H, Levesque MA, Khosravi MJ, Papanastasiou-Diamandi A, Clark GM and Diamandis EP (1996*b*) Associations between insulin-like growth factors and their binding proteins and other prognostic indicators in breast cancer. *Br J Cancer* **74**: 1242–1247

Yu H, Levesque A, Clark GM and Diamandis EP (1998) Prognostic value of prostate-specific antigen for women with breast cancer: a large United States cohort study. *Clin Cancer Res* **4**: 1489–1497

Zarghami N, Grass L and Diamandis EP (1997) Steroid hormone regulation of prostate-specific antigen gene expression in breast cancer. *Br J Cancer* **75**: 579–588

*© Cancer Research Campaign 1999*
